# Synthesis and Modification of a Natural Polymer with the Participation of Metal Nanoparticles, Study of Their Composition and Properties

**DOI:** 10.3390/polym16020264

**Published:** 2024-01-18

**Authors:** Alma Khasenovna Zhakina, Bibigul B. Rakhimova, Yevgeniy P. Vassilets, Oxana V. Arnt, Zeinulla Muldakhmetov

**Affiliations:** 1 Institute of Organic Synthesis and Coal Chemistry of the Republic of Kazakhstan, Llp., Karaganda 100008, Kazakhstan; vassilets88@mail.ru (Y.P.V.); oxana230590@mail.ru (O.V.A.); iosu@mail.ru (Z.M.); 2 Non-Commercial Joint Stock Company, Department of Biomedicine, Karaganda Medical University, Karaganda 100008, Kazakhstan; bibigul_rahimova@mail.ru

**Keywords:** polymer material, modification, humic acids, magnetite, metal nanoparticles, magnetic sorbent

## Abstract

A magnetic polymer material based on natural polymers—humic acids and magnetite, pre-configured for the sorption of a metal ion—was obtained. The magnetic polymer material was obtained via the interaction of a natural polymer, magnetite nanoparticles and sorbed metal ions that were used as a template. Moreover, the formation of a pre-polymerization complex was followed by copolycondensation with an amine in the presence of a crosslinking agent and further removal of metal ions from the crosslinked copolymer. The physicochemical properties of the resulting materials were determined using various physical methods. The composition of the resulting magnetic polymer materials was characterized by elemental analysis using an Elementar Unicube elemental analyzer. It was found that the carbon content increases by 8.28% and nitrogen by 0.42% for the polymer material Fe_3_O_4_:HA:T:AA; for the polymer material Fe_3_O_4_:HA:AA, the carbon content increases by 14.61% and nitrogen by 3.01%. Based on the IR spectra data, it is clear that magnetic polymer materials have much in common before hydrolysis (Fe_3_O_4_:HA:T:AA) and after hydrolysis (Fe_3_O_4_:HA:AA). The structure of the resulting polymer materials was studied using electron microscopy. Micrographs show the presence of pores in magnetic polymer materials after acid hydrolysis, indicating the formation of imprints. The results of the study of the sorption properties of magnetic polymer materials showed that after acid hydrolysis, the sorption capacity of a customized magnetic polymer material increases two times and it can act as a magnetic sorption material.

## 1. Introduction

The modification of natural polymers containing nanometer- and micron-sized metal particles in their composition is currently one of the intensively developing topical areas for the production of polymer materials [1,2]. The creation of such polymer materials is being actively developed at the junction of organometallic, coordination and chemistry of high-molecular compounds. This is due to the practical value of natural metal-containing polymers with a number of unique properties: high catalytic activity, unusual magnetic, electrophysical properties, biological activity, etc. [3,4,5].

In such polymer materials, the polymer matrix acts as a reactor for chemical transformations and a carrier of metal particles. The scope of application of modified metal-containing polymer materials is constantly expanding. They are used as catalysts in catalytic organic reactions [6,7]. There are known works on the use of bactericidal properties of silver- and copper-containing modified natural polymers to remove and slow down the growth of microorganisms, prevent biofouling of filtration membranes and ion exchangers [8,9]. Metal particles in modified natural polymers are used for light scattering, as additives in lubricants, and as magnetically controlled materials in analytical chemistry [10,11,12]. The search for materials based on modified natural polymers with the participation of metal nanoparticles is relevant, despite significant achievements in recent years.

Currently, the development of magnetic nanomaterials is receiving increased research attention related to the combination in the composition and the appearance of new unique physico-chemical properties in the system that allow them to be used as magnetically active materials [13,14,15]. One of the ways to obtain the magnetic characteristics of nanostructures may be to modify the surface of the obtained magnetic polymer materials. Among the promising natural polymers are humic acids—products of chemical processing of coal waste. 

Humic acids (HA) are a complex mixture of organic substances with condensed aromatic nuclei that have side chains of varying degrees of branching. The composition of macromolecules of humic acids, depending on the genesis, degree of oxidation and metamorphism, may include various functional groups: hydroxyl, carboxyl, quinone, amino, sulfogroups, etc. Coal waste from different deposits containing humic acids in their composition may differ significantly from each other in elemental composition, molecular weight, degree of condensation of molecules and the ratio of hydrophobic and hydrophilic fragments [16,17].

The special interest in natural humic acids is caused primarily by the fact that these compounds have a number of physico-chemical properties of great practical importance. Due to the presence of hydrophilic and hydrophobic fragments, they are capable of adsorption on various surfaces of the phase interface. The increase in the surface activity of natural humic acids is carried out by chemical modification, by introducing additional functional groups that make it possible to create polymer materials with fundamentally new properties. It is known that the number of functional groups in the structure of HA macromolecules is an important characteristic that determines their reactivity and physico-chemical properties [18]. In this regard, the synthesis and modification of a natural polymer with the participation of magnetite and metal nanoparticles as well as the study of their composition and properties is promising.

For the effective use of humic acids, it is necessary to consider the peculiarities of their chemical structure and composition, which determine the properties of these high-molecular natural compounds. Humic acids obtained from various humus-containing raw materials are becoming increasingly used in sorption processes as natural sorbents for wastewater treatment from various organic and inorganic ecotoxicants [19,20]. The modification of humic acids is one of the recognized methods used for increasing their reactivity. The ability of modified humic acids to bind almost all types of ecotoxicants, including transition metal ions, has been proven by studies [21,22,23,24].

Previously ref. [25] modified imprinted sorbents based on aminohumic acids with the introduction of multi-walled carbon nanotubes were created using the Molecular Imprinting method. The results of this study allow us to offer them as a sorption material with a directed sorption activity. In this regard, it is of interest to synthesize a polymer material based on modified humic acids, preconfigured for the sorption of copper ions, and to study their composition and properties.

## 2. Materials and Methods

### 2.1. Materials

The raw materials used in the synthesis of polymer material are natural polymers—humic acids (HA), a product of processing waste of oxidized coals (Kazakhstan). Natural humic acids are isolated from the waste of oxidized coals via alkaline extraction. A 0.1 M solution of sodium hydroxide was used as an extraction agent. HA was precipitated at pH = 2 using 10% HCl. They were separated by centrifugation, washed from chlorine ions and dried in a vacuum desiccator over phosphorus pentoxide. In the isolated humic acids, the yield, elemental composition and content of oxygen-containing functional groups were determined according to generally accepted methods. As a modifier, magnetite nanoparticles (Fe_3_O_4_) and acrylamide (AA) (C_3_H_5_NO, M = 71.08 g/mol, produced by Sigma-Aldrich, St. Louis, MO, USA) were used. In total, 37% aqueous formaldehyde solution was used as a crosslinking agent, and CuSO_4_·5H_2_O (GOST 19347-2014, M = 249.68 g/mol) was used as a sorbed metal ion (molecular template). The synthesis of magnetite (Fe_3_O_4_) nanoparticles was carried out according to a previously developed technique via the precipitation of Fe^2+^ salts in an alkaline medium [26].

### 2.2. Synthesis of Polymer Material Based on Modified Humic Acids and Sorbed Metal Ions

A polymer material based on modified humic acids and sorbed metal ions was carried out according to a previously developed technique [25]. The synthesis of the polymer material consisted of three stages. At the first stage, a modified pre-polymerization complex was obtained by the interaction of humic acids (HA), magnetite nanoparticles (Fe_3_O_4_) and sorbed metal ions (T). When such a modified pre-polymerization complex is formed, the polymer molecules are arranged and fixed in a certain way around the molecule of the sorbed metal ions.

The sonochemical method used in this work is based on the method described in [27,28]. The efficiency of the formation of a modified pre-polymerization complex can be improved using a preliminary modification via physical methods. The modification of the pre-polymerization complex was carried out using ultrasonic exposure. An IL 100-6/2 ultrasonic unit (Inlab, St. Petersburg, Russia) with a maximum power of 1200 W was used as an ultrasound source. The reactor was equipped with an ultrasonic generator with a magnetostrictive converter with an operating frequency of 22 kHz and a cylindrical waveguide. Under the influence of ultrasound, the processes of oxidation and reduction were enhanced. Ultrasound has a significant effect on the speed and direction of reactions, helps to increase and regulate the porous structure, change the chemical nature of the surface and improves the selectivity of chemical processes. This modified pre-polymerization complex will be called Fe_3_O_4_:HA:T.

Further, via copolycondensation of the modified complex Fe_3_O_4_:HA:T with acrylamide (AA) in the presence of a crosslinking agent, a crosslinked polymer material with a local arrangement of macromolecule sites preconfigured for the sorption of copper ions was obtained. Such an arrangement, being fixed by stitching, can be “remembered” by macromolecules, which in turn should lead to a significant improvement in the sorption properties of the polymer material. Further, this polymer material will be called Fe_3_O_4_:HA:T:AA. Removal of the molecular template (T) from the polymer mesh and formation of a polymer material (Fe_3_O_4_:HA:AA) was performed by acid hydrolysis.

### 2.3. Removal of a Molecular Template from a Crosslinked Polymer Material

Removal of copper ions from crosslinked polymer material Fe_3_O_4_:HA:T:AA was carried out by acid hydrolysis with 1 N HCl solution. To complete this, a sample of the crosslinked polymer material Fe_3_O_4_:HA:T:AA was filled with 1 N HCl solution and heated at 50 °C for 30 min. Then, the polymer material was filtered, repeatedly washed with distilled water from Cl^−^ and dried at a temperature of 120 °C to a constant weight. Further, the resulting polymer material will be called Fe_3_O_4_:HA:AA.

### 2.4. Investigation of the Sorption Properties of a Polymer Material

To study the sorption properties of the obtained polymer materials, model solutions of CuSO_4_ were used. The concentration of aqueous sulfuric acid solution varied from 50 to 150 mg/L, the pH value of the model sulfuric acids ranged from 2 to 6. The sorption of copper ions from model aqueous solutions on polymer materials was carried out in accordance with [25]. Immediately before the test, sorption polymer materials were dried at a temperature of 105–110 °C for 2 h; after cooling in a desiccator over a desiccant, they were weighed with an accuracy of 0.2 mg. Then, samples of polymer material weighing from 0.1 to 10 g were shaken with low intensity with a model solution of CuSO_4_ for 24 h. After reaching a sorption equilibrium, the polymer material was separated from the filtrate and the residual concentration of copper ions was determined in the filtrate using an atomic emission spectrometer iCAR6500 (SPECTRO Analytical Instruments Kleve, Germany) with inductively coupled plasma. The sorption capacity of polymer materials was estimated by the value of the static exchange capacity of SEC, mg/g and the sorption value R, %.

### 2.5. Investigation of the Structure and Composition of Polymer Materials

The composition of the initial humic acids and the obtained polymer materials was characterized by elemental analysis methods using the Elementar Unicube elemental analyzer (Langenselbold, Germany).

The number of oxygen-containing functional groups in polymer materials and initial humic acids was determined as follows: carboxylic—via the acetate method; the sum of carboxylic and hydroxyl groups—via ion exchange with sodium hydroxide, using the laboratory conductometer Anion-4100 (Infraspak-Analyte, Novosibirsk, Russia). The measurements were carried out sequentially on three hitches, and the average value of the three experiments was taken as the final value.

The IR spectra of the obtained polymer materials were recorded on the FSM-1201 IR Fourier spectrometer (Infraspec Company, St. Petersburg, Russia) in a wavenumber range from 400 to 4000 cm^−1^. Samples of the obtained polymer materials for this study were prepared according to the standard method of pressing with KBr. Mathematical processing was carried out using the program for curve approximation and data analysis Fityk 1.3.1.

The phase composition of the obtained polymer materials was controlled by X-ray phase analysis. X-ray images of the objects of study were recorded on a D8 ADVANCE ECHO diffractometer using an X-ray tube with a Si anode and a graphite monochromator on a diffracted beam at a scanning speed of a quantum scintillation counter—0.02 degrees/min in the area of angles 15–100° 2θ. The samples for the survey were prepared in the form of flat discs of the same mass by pressing. Phase identification, crystal structure studies and X-ray data processing were carried out using the program Bruker AXS DIFFRAC.EVA v.4.2 and the international database ICDDPDF-2 and COD (Bruker, Berlin, Germany).

Thermal analysis of the obtained polymer materials was performed on a synchronous thermogravimetric differential analyzer Perkin Elmer STA 6000 (Washington, DC, USA) in a nitrogen current, a temperature rise rate of 10°/min and a melting point up to 900 °C.

The structure of the obtained polymer materials was studied via electron microscopy. Using a MIRA 3 (TESCAN) scanning electron microscope (SEM) (Tescan Orsay Holding, Brno-Kohoutovice, Czech Republic) equipped with a system of detectors that register various signals, the particle size and surface morphology of the obtained polymer materials were estimated. Images with a topographic contrast were obtained using secondary electron detectors. The elemental composition on the surface of polymer materials was determined using X-ray energy-dispersive microanalysis.

## 3. Results and Discussion

The synthesis of the polymer material Fe_3_O_4_:HA:T:AA consisted of three stages. The synthesized polymer materials were characterized by elemental analysis methods for the attached amine, as well as by the content of the sum of oxygen-containing groups determined by conductometric titration methods. The results of this study are presented in Table 1.

As can be seen from Table 1, with the introduction of magnetite and amine into the humic acids, the carbon content increased by 8.28% and nitrogen by 0.42% for the polymer material Fe_3_O_4_:HA:T:AA; carbon content increased by 14.61%, nitrogen and by 3.01% for the polymer material Fe_3_O_4_:HA:AA. There was also a change in the oxygen-containing groups. Thus, before hydrolysis in the polymer materials Fe_3_O_4_:HA:T:AA, their content is 4.5 mg-eq/g, and ranges from 4.7 mg-eq/g upwards after hydrolysis. Therefore, acid hydrolysis increases their content. The yield of the resulting magnetic polymer material Fe_3_O_4_:HA:T:AA before hydrolysis is 86.58%, and is 80.00% after hydrolysis.

**Figure 1:**
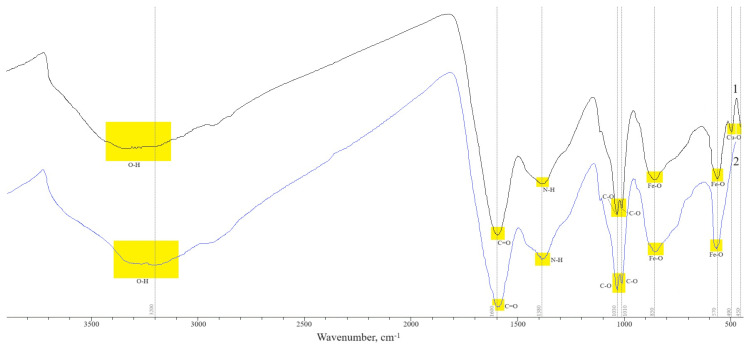
IR spectra of magnetic polymer materials: Fe_3_O_4_:HA:T:AA—1; Fe_3_O_4_:HA:AA—2.

The structure of the obtained magnetic polymer materials Fe_3_O_4_:HA:T:AA and Fe_3_O_4_:HA:AA is also confirmed by IR spectroscopy data. The IR spectra of the synthesized magnetic polymer materials are shown in Figure 1.

Profiles of IR spectra of magnetic polymer materials before hydrolysis (Fe_3_O_4_:HA:T:AA) and after hydrolysis (Fe_3_O_4_:HA:AA) have a lot in common. The spectra have identical broad absorption bands in the region of 3200–3400 cm^−1^, characteristic of O–H hydroxyl groups. Peaks at 1600–1610 cm^−1^ and 1400 cm^−1^ are caused by bending vibrations in the structure of magnetic composites of C=O and N–H bonds, respectively. The peaks at 1030 and 1000 cm^−1^ may be due to the stretching vibrations of the C–O bond in the phenolic groups of humic acids. Peaks in the region of 850 cm^−1^ and 570 cm^−1^ are associated with the magnetite phase (F_3_O_4_) [28]. The main difference in the spectra of magnetic polymer materials is observed in the region of 450–500 cm^−1^: In the spectrum of Fe_3_O_4_:HA:T:AA (before hydrolysis), there are peaks characteristic of the Cu–O bond. In the spectrum of Fe_3_O_4_:HA:AA (after hydrolysis), there are no similar peaks. This indicates the leaching of copper ions from the structure of the polymer material.

**Figure 2:**
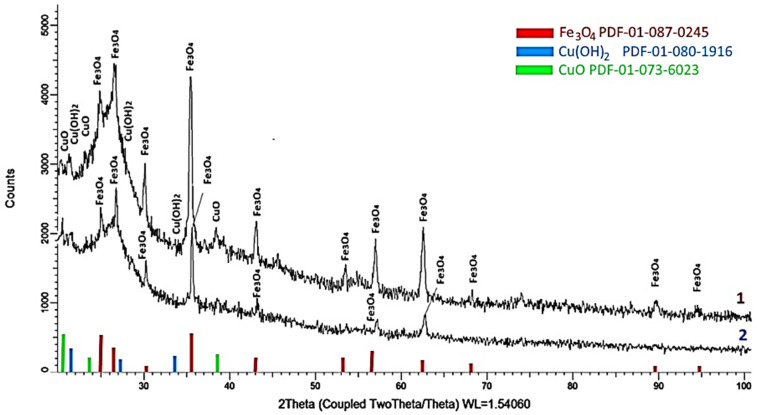
Diffractogram of magnetic polymer materials: Fe_3_O_4_:HA:T:AA—1; Fe_3_O_4_:HA:AA—2.

To determine the composition of magnetic polymer materials Fe_3_O_4_:HA:T:AA and Fe_3_O_4_:HA:AA, X-ray phase analysis was used (Figure 2).

According to X-ray diffraction data, the basis of magnetic polymer materials Fe_3_O_4_:HA:T:AA and Fe_3_O_4_:HA:AA is a mixture of two phases: the orthorhombic phase of SiO_2_ (PDF-01-073-6453) and the CaSiO_3_ monoclinic phase (PDF-01-075-1396). It should be noted that in the studied samples, in addition to the main two phases of the orthorhombic phase SiO_2_ and the monoclinic phase CaSiO_3_, inclusions in the form of a well-crystallized phase of magnetite (Fe_3_O_4_) with a cubic type of crystal structure are observed (PDF-01-087-0245), the content of which is more than 15–17%. It should be noted that these diffractograms have an almost identical shape regarding their lines and ratios.

In the sample Fe_3_O_4_:HA:T:AA (Figure 2, line 1), the formation of impurities in the form of small particles of the orthorhombic phase CuCa_2_O_3_ is observed (PDF-01-080-1066), the formation of which is due to the effects of substitution of Si ions with Cu ions, as well as the formation of oxide inclusions in the form of a monoclinic CuO phase (PDF-01-073-6023). The weight contribution of these phases is no more than 3–4 wt.% of each. 

The main difference between X-ray phase analysis of magnetic polymer material Fe_3_O_4_:HA:AA after hydrolysis is the absence of crystalline phases of CuO oxide in its composition, which indicates the removal of sorbed copper ions from the crosslinked polymer material during acid hydrolysis (Figure 2, line 2).

**Figure 3:**
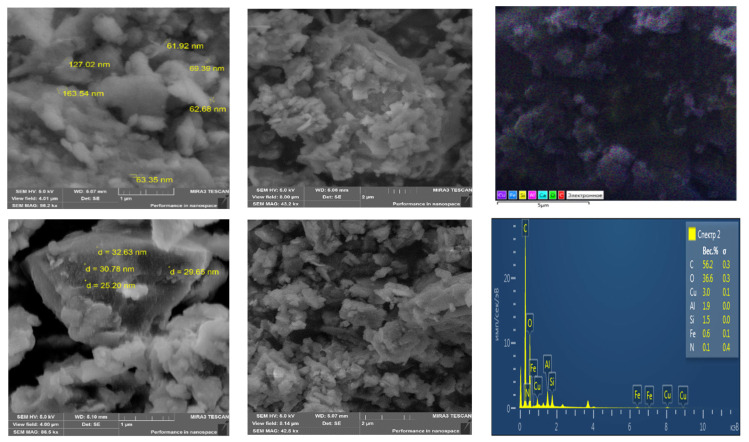
Microstructure with elemental analysis of polymer material Fe_3_O_4_:HA:T:AA.

**Figure 4:**
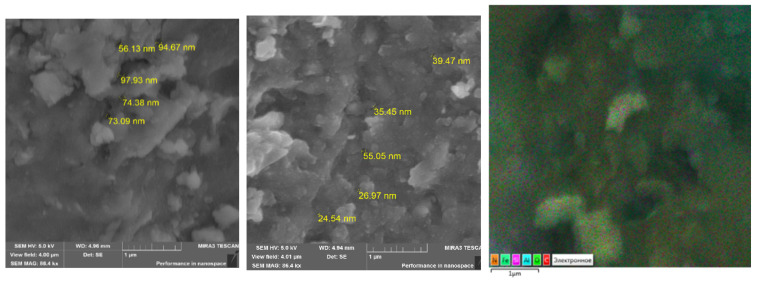

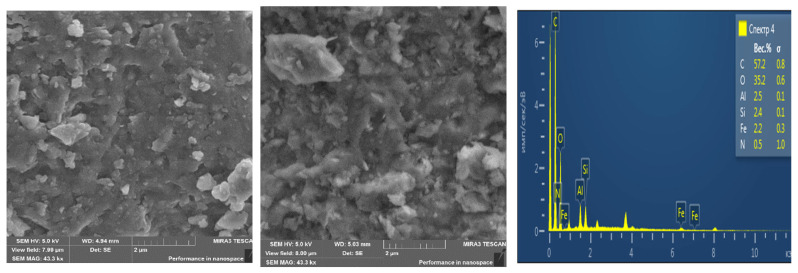
Microstructure with elemental analysis of polymer material Fe_3_O_4_:HA:AA.

Figure 3 shows topographic images of the morphology of the surface of a magnetic polymer material Fe_3_O_4_:HA:T:AA (before hydrolysis), and on Figure 4—magnetic polymer material Fe_3_O_4_:HA:AA (after hydrolysis).

The comparative analysis of micrographs of magnetic polymer materials before and after hydrolysis indicates a difference in their surface morphology and particle size. The comparative analysis of images of microstructures of polymer materials shows the differences in their surface morphology. On the electronic microphotographs of magnetic polymer material Fe_3_O_4_:HA:T:AA (Figure 3), spherical and cubic formations are visible. Particle sizes vary and range from 25 nm to 200 nm. Magnetic polymer materials Fe_3_O_4_:HA:AA (Figure 4) are characterized by increased porosity compared to magnetic polymer materials before hydrolysis (Fe_3_O_4_:HA:T:AA). Two areas of the largest pore distribution were identified for these materials. The magnification of the micrograph makes it possible to consider the distribution of pores with average diameters from 35 nm to 100 nm in the first area, and larger pores with diameters from 100 nm and above in the second area (Figure 4). Most of the pores in the magnetic polymer materials after hydrolysis were characterized by sizes close to 100 nm. This indicates that, as a result of the modification of a natural polymer with tuning, adsorption centers are formed in the polymer grid of the magnetic material, storing conformations favorable for sorption and capable of reacting with metal ions and extracting them from solution. It should be noted that acid hydrolysis washes out the sorbed metal ion and uncorks additional pores. The presence of pores with a diameter of <50 nm in polymer material Fe_3_O_4_:HA:AA is associated with voids formed after acid hydrolysis, which is clearly visible in the images recorded by scanning electron microscopy.

The analysis of energy-dispersive X-ray radiation showed that the main components of magnetic polymer materials Fe_3_O_4_:HA:T:AA and Fe_3_O_4_:HA:AA are carbon, oxygen, iron, aluminum, silicon, copper and nitrogen. Meanwhile, in the material Fe_3_O_4_:HA:AA copper ions were being washed out were pores are being released. This confirms our assumption about the release of copper from the polymer mesh after acid hydrolysis.

**Figure 5:**
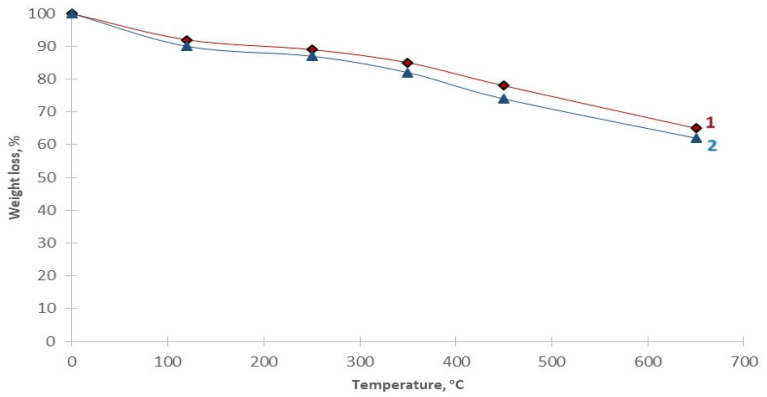
Thermogravimetric analysis curves of the obtained polymer materials: Fe_3_O_4_:HA:T:AA—1; Fe_3_O_4_:HA:AA—2.

The thermal stability of magnetic polymer materials Fe_3_O_4_:HA:T:AA and Fe_3_O_4_:HA:AA was studied using the method of differential thermal analysis (DTA). The results of this study are presented in Figure 5.

The TGA data shown in Figure 5 show a small endothermic effect and a decrease in mass in the magnetic polymer materials before hydrolysis (Fe_3_O_4_:HA:T:AA) and after hydrolysis (Fe_3_O_4_:HA:AA) in the temperature range of 90–120 °C (10 wt.%) under dynamic heating conditions. Acid hydrolysis and the release of copper ions from the polymer grid associated with the removal of hygroscopic moisture does not significantly affect the course of the decomposition curves of polymer materials. The period of the main thermal decomposition and loss of mass up to 30 wt.% starts in the temperature range above 250 °C, and the maximum speed is reached at 350 °C. This thermal decomposition is associated with the decomposition of the aliphatic components of the peripheral fragments and the organic mass of the polymer material. Total mass loss of magnetic polymer material Fe_3_O_4_:HA:T:AA is estimated at about 25–30% by weight and at about 30–35% by weight in Fe_3_O_4_:HA:AA. However, a slight loss of mass in both hydrolyzed magnetic polymer materials and non-hydrolyzed magnetic polymer materials is observed up to a temperature of 700 °C.

Previously, we [25] established the ability to selectively concentrate metal from solution in imprinted sorbents based on aminohumic acids crosslinked with “tuning”, with the introduction of multi-walled carbon nanotubes. By continuing our research in this direction, we conducted a study on the process of extracting copper ions from a model metal salt solution using magnetic polymer materials. A study of the sorption properties of a magnetic polymer material showed that, after acid hydrolysis, the sorption capacity of magnetic polymer material Fe_3_O_4_:HA:AA increase by two times compared to a similar magnetic polymer material before hydrolysis (Fe_3_O_4_:HA:T:AA). The effect of improving the sorption properties of Cu^2+^ for a magnetic polymer material Fe_3_O_4_:HA:AA is 3.6 mg/g (82.2%) and 1.7 mg/g (38.8%) for Fe_3_O_4_:HA:T:AA. Studies of the sorption properties of crosslinked magnetic polymer materials confirm our assumption that the desorption of copper ions from crosslinked tuned magnetic materials Fe_3_O_4_:HA:T:AA with 1N HCl solution leads to the formation of cavities (pores) corresponding to the ionic radius of the hydrolyzed metal and an increase in the sorption capacity of the magnetic polymer material.

## 4. Conclusions

Thus, based on the research carried out, a magnetic polymer material was obtained based on a modified natural polymer—humic acids, pre-configured for sorption by the local arrangement of sections of macromolecules. The modification of humic acids with magnetic nanoparticles and sorbed copper ions was carried out using ultrasound. The removal of sorbed copper ions from the polymer network of the magnetic material was carried out by acid hydrolysis. The composition and structure of the resulting products were proven by modern physical and chemical methods. A study of the surface morphology of magnetic polymer materials after acid hydrolysis showed the presence of pores in them, which suggests the formation of cavities (imprints) in the tuned material. The results of studying the sorption properties of pre-tuned magnetic polymer materials showed that, after acid hydrolysis, the sorption capacity of the customized magnetic polymer material increases by two times. The resulting new magnetic polymer materials composed of heavy metals have a promising use in wastewater treatment.

## Figures and Tables

**Table 1:** Yield, elemental and functional composition of polymer materials.

Sample	C, %	H, %	N, %	O, %	Yield, %	Σ(COOH + OH), mg-eq/g
HA	36.30	3.73	0.70	58.25	75.01	5.0
Fe_3_O_4_:HA:T:AA	44.58	2.83	1.13	50.42	86.58	4.5
Fe_3_O_4_:HA:AA	50.91	3.24	3.71	41.35	80.00	4.7

## Data Availability

Data are contained within the article.
